# Parents’ management of adolescent patients’ postoperative pain after discharge: A qualitative study

**DOI:** 10.1080/24740527.2020.1783524

**Published:** 2020-09-24

**Authors:** William Dagg, Paula Forgeron, Gail Macartney, Julie Chartrand

**Affiliations:** a Faculty of Health Science, School of Nursing, University of Ottawa, Ottawa, Ontario, Canada; b Faculty of Nursing, University of Prince Edward Island, Charlottetown, Prince Edward Island, Canada

**Keywords:** parental, postoperative, pain management, discharge, pediatric, adolescent, inpatient, pain

## Abstract

**Background**: Short hospital admission periods following pediatric inpatient surgery leave parents responsible for managing their child’s postoperative pain in the community following discharge. Little is known about the experiences of parents caring for their child’s postoperative pain after discharge home following inpatient surgery. Research examining parental postoperative pain management following their child’s day surgery has found that parents are challenged in their pain management knowledge and practices.

**Aims**: This interpretative phenomenological analysis study sought to understand parents’ experiences caring for their child’s postoperative pain at home.

**Methods**: Semistructured telephone interviews were conducted with seven parents between 2 weeks and 6 months after their child’s discharge from hospital.

**Results**: Identified themes were coming home without support, managing significant pain at home, and changes in the parent–child relationship.

**Conclusions**: Parents could potentially benefit from nurses optimizing educational interventions, from receiving ongoing support of transitional pain teams, and from assistance with return to school planning.

## RÉSUMÉ

**Contexte**: Les courtes périodes d’hospitalisation après une chirurgie pédiatrique en milieu hospitalier laissent les parents responsables de la prise en charge de la douleur postopératoire de leur enfant dans la communauté après son congé. On sait peu de choses sur les expériences des parents qui s’occupent de la douleur postopératoire de leur enfant après leur retour à la maison suite à une chirurgie en milieu hospitalier. La recherche portant sur la prise en charge de la douleur postopératoire par les parents suite à la chirurgie d’un jour de leur enfant a révélé que les parents font face à des difficultés en ce qui concerne leurs connaissances et leurs pratiques en matière de prise en charge de la douleur.

**Objectifs**: Cette étude d’analyse phénoménologique interprétative visait à comprendre l’expérience des parents qui s’occupent de la douleur postopératoire de leur enfant à la maison.

**Méthode**: Des entretiens téléphoniques semi-structurés ont été menés avec sept parents entre deux semaines et six mois après la sortie de l’hôpital de leur enfant.

**Résultats**: Les thèmes recensés ont été le retour à la maison sans soutien, la prise en charge d’une douleur importante à la maison et les changements dans la relation parent-enfant.

**Conclusions**: Les parents pourraient potentiellement bénéficier de l’optimisation des interventions éducatives par des infirmières, d’un soutien continu de la part des équipes de la douleur transitoire et d’une aide à la planification du retour à l’école.

## Introduction

In recent years, research has shown a decrease in length of hospital stay following inpatient surgery,^1,2^ leaving much of the recovery process to occur in the home. This is of particular concern in pediatrics because the burden of the caregiver role most often shifts to the parent.^3^ Pain management is a major concern in postoperative care, and adequate treatment of pain improves quality of life, reduces complications, and facilitates recovery.^4^ Studies have also indicated that inadequate pain management in children and adolescents can increase morbidity^5,6^ and is a risk factor for the development of chronic pain.^7^ Thus, the parental role in postoperative pain management for their child at home is of prime concern.

There is very little research exploring parents’ experiences in providing postoperative pain care for their children following inpatient surgeries (e.g., spinal fusion, colectomy), though one study examining pain at home following spinal fusion in 167 parent–patient dyads found that pain catastrophizing (a negative response to actual or anticipated pain) in children, but not parents, was correlated with postoperative pain intensity.^8^ This research also found that even at the 6-week follow-up appointment the participants continued to experience a mean pain score of 2.51 out of 10 (SD = 2.11).^8^ This suggests that not only do parents need to manage their adolescent’s pain at home for a substantial amount of time following surgery but they may also need to be knowledgeable and skilled in helping their child manage other contributing factors such as pain-related catastrophizing. A recent study by Rabbitts et al.^9^ evaluated the postoperative pain experiences of 15 parents and adolescents following spinal fusion, pectus repair, and hip osteotomy using family interviews. Findings indicated that adolescent patients and their parents were unprepared for surgery and the amount of postoperative pain the adolescent experienced, both of which contributed to a challenging recovery at home. The families in the study voiced concerns regarding potential for injury and safety, as well as stress from the adolescent’s increased reliance on parents. Though these findings are valuable for building our understanding of postdischarge pain management by parents and adolescents, the authors noted that further research was needed in the area because the study utilized a single site and almost all parental participants were female.

Much of the research on parental postoperative pain management focuses on the care provided by parents whose children underwent outpatient surgery (e.g., tonsillectomy, inguinal hernia repair, myringotomy tube insertion) and thus focuses on only a few postoperative days. Nevertheless, these studies indicate that postoperative pain management is a significant issue for parents in the home setting. Postoperative pain after day surgery can be both intense and long lasting in children, making it necessary for parents to be competent in pain management. In terms of duration of pain following day surgery, Wilson et al.^10^ examined the duration of pain in 251 patients from birth to 18 years of age undergoing head and neck surgery (e.g., adenoidectomy, myringoplasty, ankyloglossia) and found that their pain lasted on average 9 days postsurgery. In terms of pain intensity, Stanko et al.^11^ followed 100 children post-tonsillectomy to determine the incidence and intensity of pain on postoperative days 3 and 7 and found that tonsillectomy was associated with moderate to severe pain at home even into postoperative day 7. Furthermore, an integrative review of 51 articles on the pain management practices for patients undergoing tonsillectomy concluded that postoperative pain peaks on postoperative days 2 and 3 but can last up to 14 days.^12^ These findings suggest that parents of children undergoing inpatient surgeries may also need to manage pain once their child is discharged, because their child’s pain may both be intense and last for a significant period of time.

In terms of parents’ pain management practices, Fortier et al.^13^ found that parents often did not administer any medications following outpatient surgery. Parents have cited a range of concerns, including fear of drug tolerance, medication side effects, and addiction to opioids.^14^ Parents have also been found to believe that analgesics should be used as a last resort even in managing postoperative pain at home.^15^ An integrative review by Parker et al.^16^ examining parents’ postoperative pain management at home concluded that inadequate pain management was most often related to inadequate analgesic delivery and that nurses should directly target parents in their interventions. Exactly what interventions are needed to support parents in their child’s postoperative pain management in the home setting remains unclear; a study by Longard et al.^17^ found that although parents reported having their educational needs met, they still experienced difficulty managing their child’s pain at home following outpatient adenotonsillectomy surgery. Taken together these studies indicate that parents may benefit from additional resources and strategies to manage their child’s pain at home following outpatient surgery, suggesting that parents may experience similar issues following their child’s inpatient surgery.

Caring for their child’s or adolescent’s pain following outpatient surgery is challenging for parents and their pain management practices have at times been found to be ineffective. It is unclear how, or whether, these findings are different for parents providing postoperative pain to their child following inpatient surgery. Research suggests that parents may have to manage their child’s postoperative pain for extended periods of time, but most studies focus on the first 2 weeks of the postoperative period and thus it is unclear how long postoperative pain may be a problem in the home setting. This study seeks to better understand the gap in the literature by exploring the experience of parents providing at-home postoperative pain management for adolescents who have undergone inpatient surgery to provide insight into their challenges and to develop strategies to support their pain management practices in the home.

## Methods

When the topic of inquiry is the lived experiences of participants, particularly in an underexplored area, interpretative phenomenological analysis (IPA), as described by Smith and Osborn,^18^ is a valuable approach.^19^ The idiographic focus of IPA targets the unique and personal understandings of a phenomenon while accounting for the role of both the researcher and the participant within the construction of knowledge. Given the emphasis on hermeneutical ontology and the resultant social origin of truths, IPA accordingly falls within the constructivist paradigm.^20^

### Participants

Recruitment of participants occurred both prospectively and retrospectively and involved purposeful sampling to recruit parents of adolescents who underwent inpatient surgical procedures at a pediatric tertiary care hospital in Canada. Inclusion criteria were parents of adolescents who (1) were between the ages of 12 and 18 years; (2) had surgery for spinal fusion, periacetabular osteotomy, pectus excavatum repair, mammoplasty, ileostomy, colostomy, colectomy, exploratory laparotomy, femoral varus derotational osteotomy, or femur fractures; (3) were hospitalized for over 24 h postoperatively; (4) had surgery no longer than 6 months prior; and (5) spoke English. Exclusion criteria were parents of adolescents who (1) had developmental delay; (2) had a major psychiatric diagnosis; (3) experienced significant postoperative complications (e.g., deep vein thrombosis, septic infection); (4) had chronic pain diagnosis prior to surgery; or (5) were hospitalized for greater than 14 days. Exclusion criteria limited the number of possible confounding experiences influencing the adolescents’ pain experiences and thus their parents. Smith and Osborn^18^ assert that sample sizes for phenomenological studies using IPA generally range from 5 to 18 individuals but are not predetermined and are based on depth of data collected. The sample of seven parents provided rich data with sufficient depth to capture the experiences of parents caring for their adolescent’s postoperative pain at home following inpatient surgery. Therefore, no additional parents were approached to participate.

Recruitment occurred through the orthopedics team and the Acute Pain Service, where the nurse practitioners for each service introduced the study. For prospective recruitment, the nurse practitioners provided an introductory study letter and obtained approval for the research team to contact the parents, at which point the researchers provided more written information about the study. Written consent was obtained from parents in hospital during the postoperative admission or at the follow-up appointment. For retrospective recruitment the introductory study letter was mailed to parents whose adolescent had undergone surgery within the last 6 months. The introductory study letter included instructions for contacting the research team if the parent was interested in more information and in possible participation in the study. Information and consent forms were sent to interested parents via e-mail and written consent was returned via e-mail. Regardless of recruitment approach, verbal consent was confirmed by the researcher conducting the interviews by telephone immediately before the initiation of data collection. Assurances were provided to participants in the introductory letter and in the study information and consent form that the health care team would not be informed if they participated or did not participate in the study. Participants were assured that their participation in the study would not affect their relationship with the hospital. From prospective recruitment efforts, six parents were approached and five participated. From retrospective recruitment efforts, 12 letters were mailed out to potential participants, and two out of two respondents participated.

### Procedure

Once written consent was obtained by the research team, either during inpatient stay, at follow-up, or by e-mail, a time to conduct the interview was arranged no sooner than after their child’s 2-week follow-up appointment after discharge. This time frame ensured that the pain management course was not altered by serious postoperative complications and allowed for the adolescent’s postoperative pain to have peaked and perhaps be declining at the time of the interview, providing parents with a more comprehensive pain management experience.

All interviews took place over telephone and were audio recorded, allowing for the participation of parents who lived a significant distance from the hospital. A semistructured interview guide was used and included probes to allow the exploration of areas of concern and interest. In order to build trust and rapport, interview questions moved from the general to specific and from less to more personal.^18^ For example, interview questions ranged from “Can you tell me a little bit about yourself and your child?” to “What was the most difficult part of managing your child’s pain at home after surgery?” The interview guide was piloted with a parent of a healthy adolescent beforehand to determine whether the questions were understandable and that the interview flowed in a logical manner. No changes to the interview guide were required following this pilot. A professional transcriptionist transcribed each of the interviews.

### Analysis

Smith and Osborns’^18^ IPA methodology, which has a four-step process for thematic analysis that involved an ongoing and interpretative relationship with the data, was used to guide the study. The first step included a close reading of the transcript while listening to the interview to ensure accuracy of the transcripts as well as attending to the verbal nuances of the interview. Step 2 involved the noting and classifying of significant or interesting responses with a word or phrase (code). A code list was developed in collaboration with the second author after the coding of the first two interviews. However, during coding if new codes were needed, as more data were collected, earlier transcripts were reexamined with attention to the new code. Step 3 involved the grouping of codes into emergent themes, which were analyzed for each participant to ensure an idiographic (personal and unique) understanding of their experience. Step 4 involved collaboration with the research team as the emergent themes for all transcripts were compiled into larger, major themes to help identify experiences across the participants, including exemplar quotes from the data. Exemplar quotes were reviewed to ensure that the analysis was grounded in the data with attention to representation of voice among the participants.

### Rigor

In order to enhance the “goodness” of this study as per Tobin and Begley,^21^ six elements were taken into consideration in the analysis and discussion of this research: the foundation (the philosophic grounding of the research), the approach (the methodologic soundness of the study), the collection of the data (explicit reference to the means of collecting data and making decisions regarding it), the representation of voice (reflection of the both the voices of the researcher and of the participants), the art of making meaning (the presentation of insights through the interpretation of the data), and the implication for professional practice (the recommendations implied from the analysis). For example, a constructivist epistemology underpinned the interpretative nature of the phenomenological data analysis, each step in the recruitment and data collection steps have been made explicit, and the inclusion of more than one researcher during the analysis helped to ensure that no participant’s voice was dominant within the representation of the data.

### Ethics

This research was approved by the Research Ethics Boards of the Children’s Hospital of Eastern Ontario (REB approval number 18/25X) and the University of Ottawa (REB approval number H-08-18-905). Informed consent was obtained from participants by the researcher before commencing data collection. Data were de-identified at the time of transcription by removing all names and identifying information. Participants were given a study number and pseudonyms. The study number was used on the transcripts and collating of data. Pseudonyms used in the Results section further protect participant identity yet provide the reader with a contextual understanding of the data.

## Results

Seven participants were recruited; participants included five females and two males (see Table 1 for demographic details). Length of admission of the adolescents ranged from 4 days to 13 days. Interviews lasted between 30 and 60 min and were conducted between September 2018 and March 2019.

**Table 1.** Participant demographics.

Pseudonym	Participant sex	Participant age	Child’s age	Child’s sex	Surgery type
Anne	Female	41	13	Female	Spinal fusion, idiopathic scoliosis
Dana	Female	48	15	Female	Spinal fusion, idiopathic scoliosis
Frank	Male	54	16	Male	Spinal fusion, idiopathic scoliosis
Hannah	Female	54	17	Female	Spinal fusion, idiopathic scoliosis
Lauren	Female	49	16	Female	Spinal fusion, scoliosis related to muscular dystrophy
John	Male	46	17	Male	Bilateral open femur fracture reduction
Mary	Female	47	16	Male	Pectus excavatum repair

Through the thematic analysis process, three major themes were identified: *home without support: transitioning from hospital to home; we just had to deal with it: the experience of caring for a child’s pain at home*; and *it was like teamwork: changes in the parent–child relationship*. These themes illustrate the challenges encountered by participants as they cared for their adolescent child postdischarge. These themes, however, are to be viewed as abstractions and interpretative delineations made by the research team in order to better communicate and illustrate the idiographic and holistic experiences of the participants to the reader, and thus intersections and overlaps between themes exist.

### Home without Support: Transitioning from Hospital to Home

A distinctive transition phase occurred between being discharged from the hospital and arriving home. Parents spoke of wariness and anxiety in transitioning from hospital to home, with many parents feeling as though their child was too unwell for discharge, citing their child’s inability to eat or drink or independently attend to activities of daily living. Hannah described this wariness: “I would have liked just to stay one more day because the day we left with her on the Friday she was still, I mean she still couldn’t do anything on her own.” Moreover, others shared feeling as though they were being forced to leave the hospital to make room for another patient, describing discharge as dependent on the number of days postsurgery and not their child’s stage of recovery, as illustrated by Dana’s experience:

The day to leave was on Thursday and on the Wednesday, she was still very weak, very nauseous, not eating, I kept telling the nurses and the doctors, can we stay another day if she needs to stay. So I just felt a lot of pressure and I think that’s how it is in hospitals for her to leave, but I’m just saying for other parents out there, just a few days after surgery is quite fast for such a major surgery to ask them to leave, so I guess you know that’s how it is.

Furthering the difficulties parents experienced during the transition from hospital to home was the added responsibility of their child’s pain management, which was different in hospital where all pain care was provided by the nursing staff, as described by Lauren, who stated, “Oh, in the hospital I didn’t do anything.” Not surprising, parents felt unprepared to take on their new responsibilities. Moreover, feeling forced to leave and not being involved in their child’s pain care while in hospital contributed to their feelings of being alone, as Dana expressed: “You come home and you don’t have any support.”

Participants spoke of the varying degrees of educational preparation they were given prior to discharge; some participants reported receiving written instructions and others did not. Those who reported receiving written information said that the information was almost entirely focused on pharmacologic interventions, with no information given regarding psychological, social, or physical approaches to pain control or when to seek further help for their child’s pain, as voiced here by Mary and echoed by others: “We received something, I don’t remember exactly what it was, but I remember that what we received was for each medication what it does.”

It was not solely the parents’ feeling their lack of readiness to care for their child at home that was challenging but also their child’s difficulties in resuming their activities of daily living. Parents described their child’s inability to resume their normal presurgery life with significant distress, with Hannah stating,

She couldn’t wear a belt, she couldn’t bend over, she couldn’t ride a bike, she couldn’t ride a bus, she couldn’t swim, she has numbness in her feet, burning, her nerves were numb down her legs. Yeah, it was horrible. She couldn’t wake up on time, it took her long time to get out of bed, so I had to phone her high school.

Pain interfering with returning to school was an unanticipated challenge for most parents. Lauren noted that after almost 6 months postoperative, her daughter frequently needed to come home from school early, because her pain was unmanageable, stating, “There’s been times at school where she had to call home and have her dad come and pick her up because she just can’t sit anymore and there’s no place to lie down at school. … I think it frustrates her.” Being unprepared for their child’s slower than expected return to activities of daily living created an environment of significant distress because parents were unsure how to best help their child in pain.

### We Just Had to Deal with It: The Experience of Caring for a Child’s Pain at Home

Most study participants reported that their child experienced severe and significant pain, with Dana stating, “The pain was unbearable for her and she couldn’t sleep, so we just had to deal with it.” Being at home with their children was distressing for these parents because although they were aware that their child would experience pain, some parents were unprepared for the severity of pain that their children experienced at home. One parent, John, described his child’s pain crisis on return home: “I pulled in the garage and he went to get out of the vehicle and his calf muscle in the right leg spasmed and it was the first time that I heard him scream in pain in this whole situation.” In addition to parents being surprised by the intensity of pain their child experienced at home, they also spoke of the duration of pain, with most parents reporting that their child’s pain was still occurring at the time of the interview, which was between 2 months to 6 months postdischarge. Intense pain continued to the point that some of the adolescents required their opiate prescriptions to be extended beyond the initial prescription, as Dana shares here: “It took a long time. We were expecting her to be off the morphine after, you know, an extra week, but it wasn’t possible because she was still in a lot of pain overnight. In the fourth week, she was still taking the morphine.” Despite most participants voicing that their child’s pain was both more intense and lasted longer than they expected this was not uniformly experienced. For example, Anne voiced that her daughter’s pain only lasted 4 days and required no opioid analgesic postdischarge. Similarly, Frank shared that his son’s pain lasted 2 weeks and he only received morphine for the first 2 days following discharge, illustrating the individual nature of pain.

Parents reported limited experience managing children’s pain at home. Only one of the parents had previous experience in managing their child’s postsurgical pain. However, as described here by Lauren, her previous experience did not necessarily prepare her to care for her daughter after this surgery; she stated of the prior surgery, “She would just ask for Advil [ibuprofen] and/or Tylenol [acetaminophen] together, to sleep through the night she would take morphine. … I don’t believe she had dealt with pain like she had with this surgery.” Many of the parents’ stories spoke of not having the skills and knowledge to manage their child’s pain effectively, as reflected in Hannah’s story here: “I was, you know, trying to be there for her and trying to support her as much as I could, but sometimes there was nothing that we could do.” Mary, whose child had a pectus excavatum repair, described the distress she experienced when she could not protect her son from pain: “I was feeling helpless when he was in pain and I couldn’t do more for him.”

When parents described their pain management strategies, they mostly spoke of analgesic administration. Although parents administered both opiate and non-opiate analgesics, many were instructed to stop opiate analgesics as soon as possible, based more on time than adolescent need, as described here by Dana: “We were told to stop the morphine on that weekend and we just kept going.”

Parents were creative in their development of strategies to ensure the safe administration of their child’s analgesics. Two of the parents (John and Lauren) described how they created their own documentation process to track medication administrations, such as a paper chart or whiteboard. These parents voiced how they needed some way to ensure that they were administering the analgesics safely and felt that this sort of helpful strategy should be included in the postoperative instructions for parents in the future. As Lauren described, “She came home with quite a bit of medications to the point where I had a whiteboard and had to write, like, the schedule of everything just so that I didn’t mess something up or forget something or give her too much of something else.”

Parents voiced concerns about analgesics, especially opioids. Parents voiced a number of concerns regarding caring for their child’s postoperative pain. Opiate analgesics were a common concern, with parents voicing concerns such as addiction and side effects. Some, but not all parents, described trying to have their adolescent discontinue analgesics as soon as possible, with Dana stating, “I know the opioids, you know, I don’t like giving her those kind of meds knowing what’s going on out there in society.” Some parents were interested in, or had a preference for, natural and alternative remedies, speaking positively of interventions such as cannabidiol (CBD) but negatively of opiates and other pharmacologic interventions. This tendency was described by Dana but echoed by others, “I’m wondering about CBD oil for children for pain management and for nausea hum, and if hospitals should look into it in terms of you know for surgery for children, it’s a good pain management remedy apparently and there is no side effect as far as people know.”

Not all parents reported using psychological, social, and physical interventions such as guided imagery, positioning, heat packs, social support, etc. Many parents did not use formal methods of pain assessment, often opting to allow the adolescent to ask for medication if they felt they needed breakthrough pain control and using this as a marker of the presence and severity of pain, with John explaining, “Well I didn’t ask him the pain scale because if he said he needed the pain pills, as a parent I didn’t care if it was ten or a four or I didn’t really care, he was in pain, so if he said he needed something I gave it to him.”

A comment that arose multiple times was the perception of the adolescents as “brave” or “tough,” with Anne stating, “I think she was good at managing the pain, she was brave I think.” Dana described a similar sentiment, stating, “She was a trooper throughout everything and I felt that she, you know, she was gonna get through it.” These comments of “tough” or “brave” may express underlying parental or societal perceptions of pain as being unavoidable and needing to be faced and endured with strength rather than appropriately managed and thus could have contributed to poorer pain outcomes for adolescents.

### It Was like Teamwork: Changes in the Parent–Child Relationship

The process of caring for a child at home following inpatient surgery involves the changing of roles and responsibilities for both parents and adolescents. This is particularly noteworthy because a key part of adolescence is the development of independence, self-reliance, and autonomy. Frank described his 16-year-old son’s need for independence as a significant barrier in his own capacity to provide care, stating, “He doesn’t like to talk with us, me and his mother; when we give him advice or something he’s upset right away, especially from us, when, you know, when he talks with the nurse he’s a different person.” Some of the adolescents were forced into a state of higher dependence on their parents due to the severity of their symptoms and their parents were required to adapt to a higher degree of responsibility. This change was noted to be stressful because parents had been trying to support their child to take on more responsibility prior to their surgery. The degree to which this increase in dependence occurred varied among the participants. Nevertheless, all participants experienced their child’s increased reliance on them, with some of the adolescents becoming completely reliant on their parents for managing their pain management (e.g., medications, positioning), helping them dress, and other activities of daily living, as Hannah explained: “We still had to you know, help her get in her bed, lay her down, shift her body because she still couldn’t do all that. … I’m gonna say, she needed our assistance at home for a good month.” Meanwhile, other parents spoke of their child’s independence, with Lauren stating, “When she got home, she knew all her meds and what they did so she would get them on her scheduled time unless she needed like some morphine or whatever, but she asks for that, I wouldn’t offer, like I just wait for her, ’cause she knows what she needs.”

Many of the parents also spoke of a shared decision-making model with their child. In cases with a more shared decision-making process, parents directed scheduled medications and adolescents directed the need for breakthrough medications or interventions and would jointly discuss when to discontinue certain medications. Mary described her collaboration with her son when deciding to discontinue medications, explaining, “It was amongst us, like we discussed it, like when we stopped using the morphine, we had discussed it previously, but it was like teamwork, not a dictator.” Despite this, parents also spoke of the tenuous balance of supporting their child’s autonomy while concurrently describing stress in feeling as though they sometimes needed to override the adolescent’s autonomy in order to ensure that they were receiving adequate pain management. For example, Hannah discussed how she had to override her daughter’s decision not to take medications though she was visibly in pain, stating, “I mean, it was always kind of her responsibility because she is 17 and, you know, she can make her own medical decision but you know what sometimes, when the kids aren’t rationalizing, [you have to say] ‘No you should take that,’” which was echoed by other parents.

## Discussion

Parents described the experience of managing their child’s pain postoperatively through the themes of *home without support: transitioning from hospital to home; we just had to deal with it: the experience of caring for a child’s pain at home*; and *it was like teamwork: changes in the parent–child relationship*. Similar to other studies, most parents in this study reported that their child experienced severe pain following discharge^11,22^ and described feeling unprepared for their child’s discharge as well as the intensity and duration of their child’s pain.^9^

Unfortunately, our findings continue to align with studies that have discovered that many parents hold misconceptions surrounding the use of opioids.^13,14,23^ Myths and misconceptions, potentially partially underpinned by the current media landscape, may have ultimately culminated in some parents administering fewer opiates than required to adequately control their adolescent’s pain. Standardized accurate messaging from clinicians about opioids and other analgesics is still needed.

Five of the seven parents reported the duration of their child’s pain to be in keeping with the definition of chronic postsurgical pain, which is pain that lasts greater than 3 months postoperative.^24,25^ Despite this, none of the parents reported receiving a referral to a transitional pain clinic or chronic pain services or advice on treating this type of pain despite all adolescents having ongoing follow-up appointments. Transitional pain clinics can help in the pain-specific management and monitoring of patients in the ongoing postoperative period^26^ while providing access to a range of key related professional resources, including physiotherapy, psychology, and addiction services as required, thus improving the pain outcomes of patients.^27^

Many parents reported that their child experienced difficulty in returning to school due to ongoing pain but that return to school was not part of their discharge planning. Research has demonstrated that adolescents experiencing chronic pain have significantly reduced school attendance, which in turn may lead to poorer academic performance,^28^ but the rate of school attendance within the postoperative population is not known. Academic interference from postoperative pain warrants further research, including examining the effect of involving teachers and parents in a return-to-school plan for adolescents undergoing surgery.

Participants in this study reported anxiety and powerlessness related to their ineffective advocacy to delay discharge, feeling a lack of support from clinicians once they were home, and the increased demands their child’s treatment placed on them. As a result of these experiences, the parents in this study, like in other research, were stressed because they questioned their ability to care for their child.^29^ Family-centered care is a philosophy that has been purported to guide pediatric health care, which in practice should involve the empowerment of parents to provide care for their children following discharge,^30^ yet the transition from the hospital to the home was perceived by parents as clinician and time dependent rather than parent and/or child readiness dependent. Hart and Swenty^31^ described successful transition as “transition-specific skill acquisition, increased self-efficacy and self-confidence, decreased anxiety and positive coping, role mastery, sense of well-being, and identity reformation” (p. 184). Given this definition, the experiences of the parents in this study suggest that most did not experience a successful transition to home. Enhanced Recovery After Surgery initiatives have been shown to improve patient outcomes in the postoperative period through engagement of patients and families in pre-, peri-, and postoperative education and collaborative planning and goal setting^32,33^ and may aid parents and adolescents in making a successful transition.

In terms of implications, the findings from this research indicate that discharge planning needs to be a priority for clinicians caring for parents and their children undergoing inpatient surgery. Similar to Rabbitts et al.,^9^ the findings suggest that parents have an interest in gaining more in-depth information regarding postoperative recovery, including pain management (including pharmacologic, physical, psychological, and social approaches) and potential outcomes. Parents may benefit from receiving comprehensive written education regarding pain management.^34^ Parents also spoke of adolescents playing a major role in their own pain management; thus, interventions specifically targeting adolescents need to be examined. Future interventions could leverage web-based platforms to provide a source of reliable evidence-based approaches readily available around the clock with an ability to contact a clinician to enhance the transition to the home. Parents may also benefit from other styles of novel intervention, such as YouTube videos, web pages, and smartphone applications. For example, studies have demonstrated the effectiveness of YouTube videos as a method of knowledge translation targeting parental pain management strategies,^35^ explored the utility of web pages as a means of patient pain education,^36^ and demonstrated the effectiveness of a smartphone application (Pain Squad+) in engaging adolescents in their own pain management processes.^37^

There are several limitations to this study. Firstly, the children of the parents in this study all received care at a single pediatric surgical center, and it is unclear whether their experiences would be different at another hospital. However, the participants included those whose children were managed by different services, surgeons, and nurses to help improve the representation of others in this study. Secondly, most of the parents were mothers and so this study may be more transferable to the experiences of mothers than those of fathers. Research has shown that mothers and fathers differ in their interactions with children during cold-pressor tests^38^ and thus further research could explore the differences in postoperative pain management practices between fathers and mothers. Nevertheless, the experiences of the two fathers who participated did not differ from those of the mothers in this study. Thirdly, five of seven participants had children undergoing spinal fusions, which may limit transferability of findings to other populations. However, the experiences of the other two parents whose children had different surgeries were similar to those of the parents whose children underwent spinal fusions.

## Conclusion

Parents experienced many challenges in caring for their child’s pain following discharge after inpatient surgery. They spoke of being on their own and feeling unprepared to manage their child’s pain at discharge, with many speaking of the desire for a longer inpatient recovery prior to discharge. Parents noted that their children experienced varying degrees of intensity and duration of pain. Clinicians need to prepare and support parents in their pain management role. In addition to traditional educational approaches, parents could benefit from novel educational interventions with accessible instructions that they can refer to at home, including physical, psychosocial, social, and pharmacological pain management approaches. Novel approaches may include ongoing web-based coaching or online interactive sites for pain management strategies. Clinicians need to be ready to refer adolescents with difficult-to-manage pain or prolonged pain to a transitional pain clinic. Research is needed to determine the right form and timing of novel approaches to supplement traditional educational approaches in order to support parents to care for their child’s postoperative pain at home.
